# Mouse Models Reveal Role of T-Cytotoxic and T-Reg Cells in Immune Response to Influenza: Implications for Vaccine Design

**DOI:** 10.3390/v11010052

**Published:** 2019-01-11

**Authors:** Stewart Sell, Karl Kai McKinstry, Tara M. Strutt

**Affiliations:** 1 New York State Department of Health, Wadsworth Center, Empire State Plaza, Albany, NY 12201, USA; 2 School of Public Health, University at Albany, Rensselaer, NY 12144, USA; 3 Albany College of Pharmacy and Health Sciences, Albany, NY 12208, USA; 4 Burnett School of Biomedical Sciences, University of Central Florida Medical School, Orlando, FL 32816, USA; Kai.McKinstry@ucf.edu (K.K.M.); Tara.Strutt@ucf.edu (T.M.S.)

**Keywords:** influenza, T-cell cytoxicity, viral exanthema, iBALT, epithelial proliferation, mouse models, influenza vaccination

## Abstract

Immunopathologic examination of the lungs of mouse models of experimental influenza virus infection provides new insights into the immune response in this disease. First, there is rapidly developing perivascular and peribronchial infiltration of the lung with T-cells. This is followed by invasion of T-cells into the bronchiolar epithelium, and separation of epithelial cells from each other and from the basement membrane leading to defoliation of the bronchial epithelium. The intraepithelial reaction may involve either CD8 or CD4 T-cytotoxic cells and is analogous to a viral exanthema of the skin, such as measles and smallpox, which occur when the immune response against these infections is activated and the infected cells are attacked by T-cytotoxic cells. Then there is formation of B-cell follicles adjacent to bronchi, i.e., induced bronchial associated lymphoid tissue (iBALT). iBALT reacts like the cortex of a lymph node and is a site for a local immune response not only to the original viral infection, but also related viral infections (heterologous immunity). Proliferation of Type II pneumocytes and/or terminal bronchial epithelial cells may extend into the adjacent lung leading to large zones filled with tumor-like epithelial cells. The effective killing of influenza virus infected epithelial cells by T-cytotoxic cells and induction of iBALT suggests that adding the induction of these components might greatly increase the efficacy of influenza vaccination.

## 1. Introduction

Multicolor flow cytometry has revolutionized analysis of the components of protective immune responses. However, flow cytometry alone fails to capture important aspects of the interactions between immune cells and the tissues they respond in, and the process of immunopathology and/or repair taking place. Although often used simply to provide a basis of scoring the degree of inflammation associated with responses against pathogens, histological examination can be a powerful tool to reveal novel insight into mechanisms underlying health and disease that cannot be appreciated through even sophisticated flow cytometry approaches alone. In this review, we will briefly discuss how studies utilizing five mouse models of influenza permit dissection of the different components of the immune response in experimentally induced influenza infection [1] (summarized in Table 1).

Mouse models of influenza are widely used in influenza immunology research. One strength of this translational model is that the pathology of viral pneumonia is similar to humans (as will be discussed). Additional benefits of a wealth of available research tools, transgenic strains, as well as gene deficient animals far outweigh the well-recognized and acknowledged caveats of the model [2,3]. The mouse models reviewed herein have provided valuable insight into the immunopathological events in the lung resultant from viral infection that would otherwise be difficult to ascertain. Commonly used laboratory strains of mouse-adapted strains of influenza A viruses were used in these studies, and in all models the virus was administered intranasally in order to replicate as best as possible lung infection in humans. We performed blinded histological analysis of 6–8 animals per group per timepoint, examining several non-serial sections per mouse. Grading of inflammation in these models was based on both the nature of the lesion and the degree of involvement [1], and all differences among the histology scoring data were determined by the Mann-Whitney U non-parametric test. Of course, caution must be used when extrapolating the results of any model to the human condition. For example, the strains of mice used in these studies do not carry a functional Mx1 gene, which greatly increases their susceptibility to influenza infection by limiting the protective potential of the type I interferon response [4].

In the first two models, memory CD4 T cells specific for influenza were passively transferred to either wild-type (WT) or to Severe Combined Immunodeficient (SCID) mice that lack adaptive immune cells. The adoptive hosts were challenged with virus to investigate the mechanisms by which memory CD4 T cells participate in clearing infection. These studies reveal a role for cytotoxic CD4 T-cells in elimination of virus infected bronchial epithelium and type II pneumocytes [5]. In the third model, the role of the immunosuppressive cytokine IL-10 was studied during infection by comparing responses in WT or IL-10-deficient mice following influenza infection. This analysis clearly reveals an important role for CD8 T cells in the response [6]. In the fourth model, analysis of influenza-primed CCR5^−/−^CXCR3^−/−^ mice, that develop improved CD8 T cell memory against influenza, reveals not only increased T-cell mediated cytotoxicity against infected cells, but also increased BALT formation and epithelial proliferation [7]. Finally, the fifth model we will discuss addressed the role of CD4^+^ FoxP3^+^ regulatory cells (Treg) during influenza infection by treating WT mice with anti-CD25 antibody (clone PC62) to deplete this subset prior to infection. This work clearly demonstrates increased inflammation, epithelial cell toxicity, greater induced bronchial associated lymphoid tissue (iBALT) formation and markedly increased proliferation of bronchial epithelial cells and type II pneumocytes in the absence of Tregs [8]. This and findings from model 2 (SCID mice) indicate the surprising possibility that progressive lung epithelial proliferation following influenza infection may be fatal if it is not controlled by immune regulatory mechanisms. The documentation from these studies that both CD4 and CD8 cytotoxic T cells (CTL) are highly effective at clearing infected epithelial cells, and the striking induction of iBALT post-infection suggest that induction of robust CTL responses and iBALT formation should be added to the design of effective influenza vaccines. Here, we will discuss key results from our studies in relation to their bearing on the design of vaccines based on insights gained from detailed histological investigations.

## 2. A More Extensive Overview of the Results of These Five Models

### 2.1. Models 1 and 2: Transfer of CD4 T Memory Cells

CD8 T cells are considered the major subset of antigen-specific immune cells with cytotoxic potential, but recent studies have clearly identified that major histocompatibility complex (MHC) class II restricted CD4 T cells may also be cytotoxic [9,10,11]. Memory CD4 T cells provide potent secondary immunity to influenza infection in mice [12], but the scope of the antiviral mechanisms that they employ is still not clear. We showed that passive transfer of Th1-polarized memory CD4 T cells to unprimed wild type (WT) mice and to B and T cell-deficient SCID mice protects against otherwise lethal challenge doses of a highly pathogenic murine influenza virus (A/PR8) by attacking infected epithelial cells in the lung [5]. Grading of inflammation in this model, as in the other models that will be discussed, is based on both the nature of the lesion and the degree of involvement [1].

#### 2.1.1. Model 1: Transfer of CD4 Memory T Cells to Unprimed WT Mice

Unprimed WT BALB/c mice received the same number of either naive or memory transgenic CD4 T cells that express a T cell receptor for an epitope of the A/PR8 influenza virus (referred to as HNT transgenics) by intravenous injection. The same day the recipients were challenged with a dose of 10,000 EID_50_ of the A/PR8 that is lethal to mice not receiving memory HNT cells, but against which the transfer of HNT cells protects. After considering the ‘take’ of the donor cells, this transfer reconstitutes mice with a quantity of cells that is consistent with the total number of virus-specific memory CD4 T cells that can be detected in an influenza-primed mouse [5]. Our goal was to test the protective capacity of memory CD4 T cells against influenza in the absence of any other immune cells primed by the virus, and to determine the mechanisms that these cells employ to combat infection [5,13].

**Figure 1:**
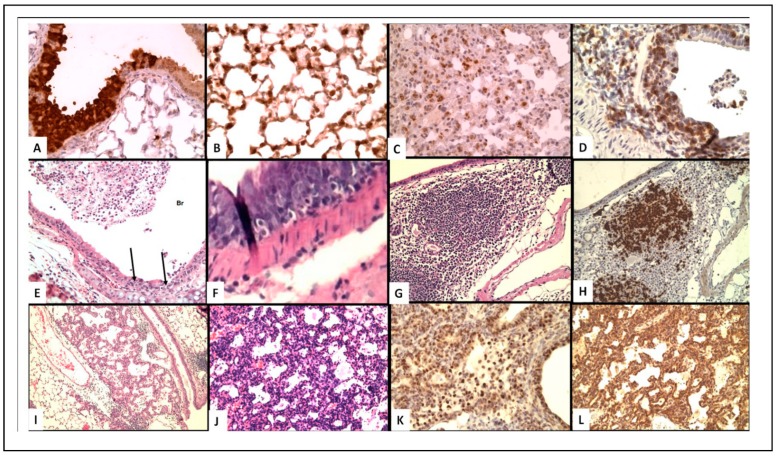
(**A**–**C**) Virus localization in epithelial cells. (**D**–**F**) T-Cell infiltrate in epithelial cells. (**G**,**H**) iBALT (**I**–**L**) Epithelial proliferation. (**A**) Immunoperoxidase staining of influenza virus in bronchial epithelium of WT mouse at 8 days post-infection (200×); (**B**) Virus in type II pneumocytes in alveoli of WT mouse (400×); (**C**) Decreased virus staining in type II pneumocytes with lymphocytic infiltrate in mice receiving memory T cells (400×); (**D**) CD3 T-cell staining of peri-bronchial T-cells and cells in bronchus of SCID mice 1 week after transfer of memory CD4 T-cells (400×); (**E**) H&E showing lymphocytes infiltration of bronchi and desquamated cells in WT mice 2 days after transfer of memory CD4 T-cells (100×); (**F**) H&E of intraepithelial lymphocytes in IL-10 knock-out (KO)mice, day 8 (400×); (**G**) H&E iBALT 5 days after secondary infection of Treg depleted mice (40×); (**H**) B-cell staining PAX5) of iBALT (100×); (**I**) H&E epithelial proliferation in SCID mice day 14 after transfer of memory CD4 T-cells (100×). (**J**–**L**) proliferating epithelial cells in Tregs depleted mice week 4; (**J**) H&E; K. surfactant; (**L**) TTF (200×).

Figure 1A,B shows virus in bronchial epithelium (Figure 1A) and type II pneumocytes at 8 days post-infection (Figure 1B), confirming acute infection in the expected cell types. The mice receiving naïve cells all die by about day 10, whereas mice receiving memory cells all survive and clear virus by day 10 (based on analysis by RT-PCR). These outcomes correlate with striking differences in several histological observations. First, there is slight perivascular and peribronchial infiltration with mononuclear cells after transfer of naïve cells, but a marked increase associated with transfer of memory CD4 T cells. Second, there is increased T-cell invasion and disruption of the bronchial wall basement membrane. Third, many more lymphocytes surround type II pneumocytes in mice receiving memory cell transfer, with reduced staining for virus, indicative of improved clearance (Figure 1C). Given that the transferred memory cells were generated in vitro under conditions not generally thought to promote cytotoxic effectors, a striking conclusion from this analysis is that the cytotoxic T-cell infiltration and reaction with infected epithelial cells (demonstrated by immune-labeling for virus) correlates with protection against lethal infection. A caveat to this analysis, however, is that the donor memory cells were not distinguished from host T cells, and thus it is difficult to ascribe any type of cytotoxic activity exclusively to the donor cells.

#### 2.1.2. Model 2: Transfer of CD4 Memory Cells to SCID Mice

To refine the above analysis and focus on the relationship between protective memory CD4 T cells and histological changes observed during protective responses against influenza, we took advantage of a transfer model using SCID hosts. As SCID mice are more susceptible to infection than are WT mice, we used a lower dose of A/PR8 virus than used in the experiments discussed above (2500 EID_50_) that is nevertheless lethal to all SCID hosts not receiving memory cells. Memory CD4 T cells promote robust reduction of viral titer in SCID mice during the first week of otherwise lethal doses of influenza, but without contributions from other components of the adaptive immune system, such as virus-specific antibody or CD8 T cells, viral titers are not fully cleared [5]. Instead, histological examination of lungs reveals extensive epithelial proliferation occurring throughout three- or four-weeks post-infection when the mice eventually die [5]. Focusing on the first week of infection, during which time the memory CD4 T cells control viral titers, our analysis revealed focal perivascular, marked interstitial, and peribronchial collections of lymphocytes. Little evidence of hyperplasia of the bronchial epithelium is observed during the first week of infection, and the lung periphery (including alveolar sacs) is relatively normal. The lymphocytic infiltrate contains only T-cells (the donor memory CD4 T cells, which represent virtually all CD3+ cells in the lungs as judged by flow cytometry) and large numbers of T-cells are in the epithelial layer (Figure 1D). The bronchial epithelium is hyperplastic in some sections but there is no evidence of continuing injury. These observations suggest that at least some of the T cell infiltration observed in WT mice discussed above are donor memory cells, and that these are likely marked by cytolytic potential. Indeed, we saw that SCID recipients of perforin-deficient memory CD4 T cells had increased viral titers compared to recipients of WT memory cells, suggesting that these infiltrating donor cells contribute to viral control through classic perforin- and granzyme B-dependent killing [5].

Histological analysis past the second week of infection also revealed unexpected findings that help to explain the cause of virus-associated death in SCID hosts, and that might suggest a novel role for memory CD4 T cells in regulating lung epithelial proliferation. By week 3, bronchial epithelial cells extend into the adjacent pulmonary parenchyma, most likely derived from focal proliferation of epithelial cells along terminal air sacs or alveolar walls with squamous metaplasia. By week 4 (Figure 1I), the degree of focal proliferation of bronchial epithelium, seen as solid collections of epithelial cells resembling small epithelial tumors, is increased further with some areas showing early squamous metaplasia. The expanding epithelial cells label for both thyroid transcription factor (TTF, stain for bronchial epithelial cells) and protein C (surfactant for Type II pneumocytes) [1]. This hyperplastic response of type II pneumocytes and/or terminal bronchial epithelial cells appears to be attempting to restore sloughed epithelium. The proliferative response will be presented in more detail in experiments to follow. Although there is a prominent peribronchial lymphocyte infiltration, organized collections of BALT as described below are not seen, most likely because iBALT formation requires a major participation of B-cells [14], which are not present in the SCID mice. The unchecked proliferation of epithelial cells could be the cause of the eventual death of these mice. Further studies are required to work out the mechanisms, but it is possible that these observations reflect reparative processes initiated by memory CD4 T cells that under conditions of acute infection are beneficial, but in this chronic model of infection become pathogenic. Such a conclusion is challenging to arrive at through flow cytometry-based analysis of the lungs, or indeed by measures of gene expression or soluble factors in the infected lung and highlight the insight that can be gained by careful histological analysis.

#### 2.1.3. Transfer of Memory CD4 T Cells to WT Mice and Intranasal Challenge with Non-Infectious Antigen (Ovalbumin)

To determine the effect of reaction of memory CD4 T-cells with soluble antigen in the lung in the absence of infection, WT C57BL/6 mice that were given memory CD4 T cells specific for ovalbumin protein (generated from OT-II transgenic mice) were challenged with ovalbumin intranasally in the absence of any kind of adjuvant signal [15]. We reasoned that this model would clearly differentiate histological changes that were driven by memory CD4 T cells recognizing and responding to antigen in the lung versus those that were driven by infection and infection-induced inflammation. 48 h after ovalbumin administration there is marked perivascular infiltration by lymphocytes with essentially no involvement of the bronchi or bronchioles, whereas no changes are observed in mice not receiving ovalbumin-specific memory cells. These rapid histological changes are consistent with rapid memory CD4 T cell-induced production of many proinflammatory cytokines and chemokines from innate immune cells within the same timeframe following intranasal ovalbumin administration or viral infection with influenza expressing ovalbumin-peptide [15]. Again, the analysis of histopathology synergizes with flow cytometry-based analysis of cellular populations and ELISA-based analysis of proinflammatory mediators to construct a more wholistic picture of pulmonary immune responses.

Although the eliciting antigen (ovalbumin) diffuses from the bronchus into the lung, the reactive T-cells migrate from the vessels so that the antigen-T-cell reaction takes place in the perivascular space resulting in the perivascular mononuclear cell reaction observed. This may be compared to a delayed hypersensitivity skin test, and the inflammation peaks at 24 to 48 h after antigen challenge. This ‘delayed hypersensitivity reaction’ in the lungs mediated by memory CD4 T cells responding to antigen does not extend to the bronchi. In contrast, WT and SCID mice receiving virus specific memory CD4 T cells prior to infection with influenza (as in the model discussed above) show marked alveolar peribronchial and bronchial inflammation, presumably in this case due to reaction of memory cells with peptide expressed on the surface of influenza-infected cells. Thus, memory CD4 T cells may mediate T-cell cytotoxicity in the lung when directed to virus infected cells or delayed-type hypersensitivity when directed to soluble protein antigens. It is still unclear if distinct subsets within the memory population mediate each of these responses, nor is it entirely clear what kinds of inflammatory signals are needed to initiate these outcomes.

### 2.2. Model 3: IL-10 Deficient Mice (Increased CD8 T-Cell Activity)

IL-10 is a pleotropic cytokine also known as cytokine synthesis inhibitory factor. In the context of infection, it is generally produced by effector CD4 and CD8 T cells, and it acts via STAT3 signaling to downregulate expression of Th1 cytokines and CD4 T-cell activation [16], but can also have a variety of other diverse roles, especially during viral infection [17]. Given these potential mechanisms of action, we challenged WT or IL-10 KO BALB/c mice with influenza to ask whether IL-10 expression would improve the outcome of infection (by reducing immunopathology associated with otherwise too aggressive immune responses), or worsen the course of disease (by unleashing a more immunopathogenic response against the virus. The lungs of WT or IL-10 KO mice were examined 6–8 days following challenge with 500 EID_50_ of A/PR8, which causes only mild disease, or with 5000 EID_50_, a dose resulting in the death of 50% of the WT mice. Interestingly, this dose, though lethal was not high enough to produce acute lethal necrotic bronchitis observed classically. In contrast to the WT mice, only 20% of the IL-10 deficient mice died demonstrating a detrimental role for IL-10 production in this model [6]. No differences in survival or the course of disease were observed between the WT and IL-10 KO mice using the lower dose of virus. These results indicated to us that IL-10 may play a detrimental role in the lung during the response against higher doses of influenza that may better mimic situations of highly pathogenic infection in humans.

Histological analysis at day 8 revealed that invasion of the bronchial epithelium by lymphocytes (Figure 1F) and destruction of epithelial cells was much greater in the IL-10 KO mice. This finding most likely reflects increased CD8 T-cell cytotoxicity, as many more cytotoxic CD8 T cells than CD4 T cells are present in the lung, given that only about 10 percent of CD4 T cells in the lung express a cytolytic phenotype [18]. Furthermore, peribronchial collections of T-cells, and extensive infiltration of the epithelial layer of the bronchus with T-cells associated with separation of epithelial cells were more pronounced in the better protected IL-10 KO mice. Very few B-cells were observed in this infiltrate and no staining of cells with F4/80 (a macrophage marker) was seen. There was no epithelial proliferation observed at this relatively early time point after infection. Together, these results indicate the role of CD8 cytotoxic T-cells in the immune reaction to influenza virus infected epithelial cells is increased in the absence of IL-10. It is important to note that we did not observe increased CD8 T cells in the lungs of IL-10 KO versus WT lungs by FACS. Histological analysis can thus help reveal discrete changes in the positioning of cells within organs under different conditions, even in cases where the total numbers of a given subset are not found to be remarkably different.

### 2.3. Model 4: CCR5^−/−^/CXCR3^−/−^ Mice (Enhanced Memory CD8 T-Cells)

The studies were conducted to determine if CD8 T cell responses to discrete chemokine signals (mediated by the receptors CCR5 and CXCR3) within the lung altered their positioning and subsequent memory fate. Indeed, improved CD8 memory was seen in mice deficient for both CCR5 and CXCR3 [7], that had major defects in development or hematopoiesis in the steady state. A model was thus established to ask whether this improved memory, or other factors associated with a loss of signaling through these chemokine receptors, could impact recall challenges to influenza. Briefly, WT and CCR5^−/−^CXCR3^−/−^ mice on a C57BL/6 background were primed with a sublethal dose (500 EID_50_) of the X31 strain of influenza (H3N2). X31 is murine influenza strain that drives milder disease and is commonly used to prime mice in order to test aspects of the immunity raised following challenge with the more pathogenic viral strain A/PR8. After 90 days, the primed mice were rechallenged with a supralethal (50 LD_50_) dose of A/PR8 (H1N1) to assess heterosubtypic immunity, which is largely mediated by virus-specific memory CD4 and CD8 T cells [19]. Interestingly, both WT and CCR5^−/−^CXCR3^−/−^ were able to clear the secondary challenge by 8 days post-infection [7].

The rechallenged mice were sacrificed at days 0, 3, 7, and 14 days and their lungs examined. This allowed for a unique investigation of how major chemokines associated with classic Th1/Tc1 responses shape protective responses against influenza, which are of interest to vaccine design. Inflammation and epithelial proliferation occurred in both WT and chemokine receptor-deficient mice group but were quantitatively much greater in the CCR5^−/−^/CXCR3^−/−^ mice [7]. This correlated with the presence of more CD8 T cells in the lungs of CCR5^−/−^/CXCR3^−/−^ mice. There was also marked mononuclear cell infiltrate around the bronchi, and extensive proliferation of epithelial cells from the terminal bronchi into the adjacent alveoli. Again, these epithelial cells stain for both TTF and protein C [1]. The majority of the periarterial and peribronchial lymphocytes seen were T-cells (CD3+) with few, if any, B-cells or macrophages, consistent with observations in the primary infection models discussed above.

One major difference in the lungs of mice undergoing primary versus secondary infection with influenza is the presence of iBALT in the latter. The prominent iBALT in primed mice stains heavily for B-cells whereas staining for T cells is scant. The presence of larger unlabeled cells suggests the presence of F4/80 negative dendritic cells or other antigen presenting cells within the iBALT. These studies suggest that modulating chemokine-based trafficking within the lung is a viable strategy to improve influenza-primed memory CD8 T cell generation, with perhaps broader than expected consequences during recall challenge. Further studies are required to determine whether the distinguishing aspects of the response in CCR5^−/−^/CXCR3^−/−^ mice are linked to improved memory CD8 T cell generation. These results also indicate that robust iBALT formation could be a critical aspect of improved vaccine strategies.

### 2.4. Model 5: Depletion of Regulatory T Cells

Finally, a protocol was established to investigate the extent to which FoxP3+ regulatory CD4 T cells impact the outcome of primary and heterosubtypic influenza infection. Again, in addition to employing other means to assess the immune response, histological changes were examined to gain a more complete picture for how Tregs modulate the host response. Wildtype C57BL/6 mice were primed with a relatively high sublethal dose of X31 virus (3000 EID_50_) and challenged after 35 days with a supralethal dose of A/PR8 (60,000 EID_50_). The contribution of Tregs to controlling the immune response during secondary influenza infection was examined in untreated mice as well as in mice treated with anti-CD25 antibody (clone PC61) prior to virus re-challenge. A single dose of 100 μg of this antibody clone given intraperitoneally preferentially depletes Tregs that express very high levels of the IL-2 receptor alpha chain (CD25) under steady state conditions [20].

Interestingly, the depletion of Tregs did not impact the survival of primed mice, nor their weight loss or efficiency of viral clearance [8]. Histological analysis of lungs revealed that depletion of Tregs resulted in significant increases in both inflammation and epithelial cell proliferation; accompanied by an increase in antigen-specific memory CD8+ T cell responses. Furthermore, the conversion of areas of the lung next to bronchi into iBALT was much greater in mice depleted of Tregs (Figure 1G,H), than in mice with Tregs, which feature only loose collections of large monocytes. Finally, in the Treg-depleted mice, large areas of the alveolar spaces were filled with epithelial cells (Figure 1J–L), which was markedly higher than that observed in Treg-replete animals. Such proliferation has previously been described during primary infection beginning at 3–6 days and peaking in the alveoli at about 2 weeks (see discussion below). The absence of Tregs thus appears to exacerbate this already robust repair process, as it expands to occupy more than 50% of the lung tissue. Additionally, some of the epithelial cells in the histologic slides demonstrated squamous metaplasia, even when sacrificed only 5 days after infection—which is quite early in the repair response (even during secondary infection) to observe such extensive proliferation. These observations thus indicate that the functional loss of Treg activity appears to allow unchecked, fatal epithelial proliferation during secondary influenza challenge [8]. This is an intriguing result as analysis of Treg function in most studies focuses on their ability to regulate the activity of other leukocytes.

## 3. Discussion

The careful histological analysis of influenza-infected lungs can identify not only differences in the degree of immunopathology between experimental and control groups of mice, but also important insights into the mechanisms of protection acting during primary and secondary infection. Below, we will briefly highlight and integrate some of the key findings from our studies. Importantly, we will also discuss a vaccination strategy against influenza that is guided by histological examination, which we believe represents a novel approach for vaccine design.

### 3.1. T-Cell Cytoxicity

In each of the models of influenza infection discussed there is clear evidence of T-cells invading the influenza infected epithelial cell layer. These observations are consistent with extensive attack on epithelial cells by cytotoxic T-cells, presumably through MHC-mediated antigen recognition. Experiments utilizing SCID hosts provide evidence that memory CD4 T cells alone can mediate this damage, which likely involves a highly specialized cytotoxic CD4 T cell subset observed in several models of influenza infection. Analysis from some of the other studies highlighted here also indicate that CD8 T cells are also involved in this process, which is as expected given the important role that CTL play in optimal viral clearance. Richard Shope first identified the transmissible agent of influenza and in 1931 described lymphocyte infiltration of bronchial epithelium using a model in which pigs were treated with material from influenza-infected human patients [21]. He described exudative bronchitis accompanied by bronchial epithelium damage, including fragmented and partially desquamated tissue, with the observation of leukocytes, found singly or in clumps. These findings are reminiscent to the lesions described in our experiments. The experiments using the transfer of CD4 cells to SCID mice infected with virus points to extensive T cell-mediated damage underlying this process, as SCID mice not receiving T cells, that die of viral-induced causes, do not show such pathology. Whether or not T cell responses against influenza can be optimized to avoid or minimize collateral damage associated with the anti-viral response is an ongoing area of investigation. We point out, however, that analysis in Model 5 indicate that priming Tregs may be an important strategy to this end.

In 1937, Straub described lymphocyte infiltration and “denudation” of bronchial epithelium in mice infected with a human influenza isolate [22]. Straub’s observations essentially match key aspects of the histological changes that we describe above. More recently, a study from Bi et al. [23] also reported lymphocyte infiltration of bronchial epithelium and its separation from the basement membrane following infection of mice with novel reassortment H9N2 viruses isolated from chickens. These findings thus appear to be consistent across diverse influenza strains, and are seen in different animal models. As such, they are encouraging as the mouse model allows for unparalleled approaches to investigate the underlying mechanisms driving both the beneficial and deleterious outcomes of T cell responses against the virus.

### 3.2. iBALT

BALT is a tertiary lymphoid structure in the lung where local immune responses can occur rapidly, and is inducible in humans and mice after inflammation [24,25] resulting in so-called inducible BALT (iBALT) [26,27]. In all the models that we analyzed in which B cells were present, we observed extensive iBALT that was induced by influenza infection. The prevalence of iBALT in relation to the models of influenza infection discussed here was increased by memory T cell transfer but decreased with the loss of IL-10. The relevance of these findings is still not clearly understood. However, the presence of iBALT in mice correlates with more rapid influenza clearance and improved survival [28,29]. Interestingly, iBALT that is formed in response to viral infection has been shown to enhance immune responses to subsequent infection with unrelated viruses (heterologous immunity), by impacting the production of local cytokines and boosting pulmonary antibody production [28]. As such, priming iBALT through vaccination may lead to improved protection against influenza, and perhaps other respiratory pathogens.

### 3.3. Epithelial Proliferation

In his seminal studies [22], Straub also reported extensive epithelial proliferation in the infected lung. His observations were later repeated and extended by Oliphant and co-workers [30,31], Taylor [32], Dubin [33], and by Loosli [34]. The marked hyperplasia and growth of bronchial epithelium into surrounding lung tissue can persist for up to 3 months, and this invasive growth implies malignant transformation [35]. Recently Qiao et al. [36] describe severe interstitial and intra-alveolar fibrosis, collapsed alveoli, and large fibrotic areas in BALB/c mice 30 days after influenza challenge. We point out that enlargements of Figure 1E,F of the Qiao paper also show epithelial proliferation with squamous metaplasia. From the studies summarized in this review it is also clear that the epithelial proliferation that occurs after the first week of influenza infection can essentially fill up the alveoli of large portions of the lung. Fortunately for the surviving mice, these proliferative lesions appear restricted to areas where infection occurred. The epithelial proliferation extends from the terminal bronchi where progenitor cells, including those giving rise to epithelial cancer, may be concentrated [37]. The proliferating cells stain for both protein C and TTF, as would be expected for putative lung epithelial progenitor cells [38]. While normally self-limiting, in SCID mice or mice with depleted of Tregs, epithelial proliferation continues for several weeks post-infection until the mice die. We speculate that this process may in fact drive death in these models, and perhaps in some clinical scenarios of highly pathogenic influenza infection (see below). These observations suggest that epithelial proliferation associated with recovery from influenza infection behaves like an immune sensitive carcinoma. Indeed, our observations of repair processes involving half of the lung tissue in mice depleted of Tregs responding to secondary influenza challenge raises concerns about the recent suggestions that depletion of Tregs might be used to enhance the immune response in cancer therapy [39].

In fatal human influenza not complicated by secondary bacterial infection there is multifocal superficial necrotizing tracheo-bronchitis, alveolar necrosis and hyaline membrane formation with edema, hyperemia and mixed mononuclear infiltration of the lamina propria [40]. The necrotizing broncho-alveolitis includes death of type I and type II pneumocytes. Examination of the cases of the 1918 epidemic revealed that the destruction of the bronchial epithelium is followed by extensive proliferation of epithelial cells [40]. Mitotic activity can begin as early as 5–14 days after the onset of infection and extends from terminal bronchioles into adjacent alveoli with loss of lung function. Since these pathologic studies were done on autopsied cases, it is likely that the proliferation contributed to the death of those examined, but this has not been definitively established and the possibility of malignant transformation has not been ruled out. Most of the tissues examined in these studies were from autopsy material of patients who died acutely from the infection. Thus, there is much less known about the pathologic changes that occur early in humans who survive. Several studies have been done on lung biopsies from patients while still alive less than 2 weeks after infection [41,42,43]. In these studies, the acute changes in the lung varied from patchy fibrinous alveolar exudates and hyaline membranes with interstitial edema to severe diffuse alveolar damage and necrosis of the bronchiolar mucosa. Also prominent were reparative changes including proliferation of epithelial cells, mild interstitial chronic inflammation and organization within air spaces and in the interstitial tissue. These findings are thus consistent with many of the processes we have characterized in the murine models discussed above, further supporting the utility of mouse models to gain insight into strategies to limit deleterious outcomes, especially those associated with the T cell response against the virus. Of course, all animal models of influenza infection have strengths and weaknesses [44], and further evidence of the correlation between the early events following infection in mice and humans is needed.

### 3.4. Bronchial Epithelial Destruction and Contact Dermatitis

A highly intriguing outcome of our analysis is that the infiltration of bronchial epithelial with cells associated with destruction of the epithelial cells observed in the lung following influenza infection shares striking similarities with the defining characteristics of skin lesions associated with contact dermatitis (i.e., poison ivy) and viral exanthema including smallpox and measles [45]. In contact dermatitis cytotoxic T-cells react with the antigen diffusing into the dermis. Because most of the lipid soluble antigen is attached to epidermal cells, in sensitized individuals it takes about 2 days for the reacting cells to invade from the dermis, so that poison ivy reactions usually peak around 48 h. Epidermal cells are separated from each other and destroyed via a form of anoikis [46] also seen in cell mediated autoimmune diseases, such as auto-allergic thyroiditis [47]. This reaction is also responsible for the skin lesions of measles, rubella, erythema infectiosum and roseola, as well as for the “take” reaction to vaccinia vaccination against smallpox [48,49]. We suggest that one approach to improve the ability of vaccines against influenza to induce protective immunity is to prime responses that are able to better mirror the kinds of histological changes seen during responses against infection. Below, we briefly summarize evidence that a Vaccinia-based influenza vaccine may represent a promising candidate to this end.

### 3.5. Relevance to Vaccination

Conventional influenza vaccines protect against infection by inducing circulating antibody that blocks the interaction of the virus with potential target cells. Every year a new seasonal vaccine is made to protect against the strains that are predicted to circulate. There are two major problems with this approach: First, the vaccine epitopes are highly mutable surface proteins. This means that there are often mis-matches between the vaccine and the prevalent circulating strains due to poor prediction. Second, although blocking antibody may prevent infection, it is likely that a vaccine that induces influenza directed CD4 and CD8 T cell responses will produce longer-lasting and more effective immunity, not only to the immunizing strain, but also to other more virulent strains than the presently used vaccines [50,51]. We will not discuss here in detail the mechanisms of protection mediated by influenza-specific T cells during recall responses as we and others have done so previously [52,53]. We point out however that protection from influenza disease correlates better with influenza-specific T-cell reactivity than antibody responses [54,55], and our results clearly show the effective role of both CD4 and CD8 cytotoxic cells in eliminating influenza virus from infected lung epithelium. As reported above, the cytotoxic immune response in the lung features intraepithelial invasion of T-cells and destruction of infected epithelium in a reaction analogous to that of the vaccination response to small pox. Vaccinia virus is the vaccine for smallpox. Thus, as suggested by our analysis of histological changes accompanying protective T cell responses against influenza, recombinant vaccinia virus-based vaccines may be effective at inducing cytotoxic T-cells, perhaps more so than presently used vaccines.

### 3.6. Vaccinia Vectors as Influenza Vaccine Candidates

Indeed, many antigens expressed by vaccinia virus vectors have generated strong antigen-specific T-cell and antibody responses. Immunization with vaccinia vectors is most effective when the antigen is presented in association with MHC class I on the surface of epidermal cells. When vaccinia infects epithelial cells the vaccinia antigens are presented by the endogenous pathway and T-cell cytotoxicity rather than delayed-type hypersensitivity is induced [56]. Indeed, the eschar formed in response to vaccinia vaccination is mediated by T-cell cytotoxicity. A study has been reported in which a DNA copy of the influenza virus hemagglutinin gene, derived from influenza virus A/Jap/305/57 (H2N2,) was inserted into the genome of vaccinia virus strain WR Vaccinia strain under the control of an early vaccinia virus promoter. Hamsters infected intradermally produced circulating antibodies that inhibited hemagglutination and were protected against respiratory infection [56]. Protection in this instance was credited to neutralizing antibody, but the generation of CD4 or CD8 T cell responses by the vaccine was not analyzed. Thus, contributions from these subsets to the protective response cannot be discounted. The WR strain virus has been used for many studies on pathogenesis (see below), however use of a less pathogenic stain for protective vaccination seems advisable. The properties of biosafety 1 level handling, avirulence, low replication rate after infection and high immunogenicity have been cited to propose recombinant modified vaccinia virus Ankara (MVA) as the vector of choice for preclinical and clinical studies [57].

In fact, evidence from both human and animal studies indicates that such Vaccinia-based vaccination against influenza can induce robust, protective immune responses. A fusion construct of nucleoprotein and matrix was found to be safe and immunogenic in chickens using MVA [58]. Furthermore, a recombinant vaccinia virus containing the PR8 viral nucleoprotein (NP) gene was able to stimulate a vigorous secondary cross-reactive CTL response, even against human influenza isolates [59]. In human clinical trials, an MVA vector encoding nucleoprotein and matrix protein 1 (MVA-NP + M1) was safe with minimal side effects and induced high frequencies of CD8+ antigen-specific T-cells as measured by IFN-γ ELISPOT assays [60]. CD4 responses were not investigated in this study. Volunteers, including elderly subjects, receiving a single vaccination with MVA-NP + M1 had increased T-cell responses to the vaccine antigens and had significantly fewer symptoms after influenza challenge than controls [61,62]. These findings support the concept that an optimized vaccinia-based vaccine strategy against IAV could induce robust, long-lived antigen-specific T cells in addition to protective antibody.

### 3.7. Vaccination Route

Another striking aspect of our observations is the development of local immunity that occurs following intranasal infection. It makes sense that an optimized vaccine against a given pathogen should target the induction of protective immune cells at the site of infection. In the case of influenza, the site of infection is the lung, but the field’s understanding of immunity at mucosal sites is still incomplete. Nevertheless, a live attenuated influenza vaccine, FLUMIST^®^, administered intranasally has been approved by the Centers for Disease Control and Prevention for the 2018–2019 influenza season after a 2-year hiatus for evaluation after changing the type A H1N1 component. As shown above intranasal challenge not only induces cytotoxic T-cells, but also formation of iBALT that serves as a local site for a secondary antibody response in the lung. As stated above vaccinia readily infects lung epithelium of mice and careful vaccinia strain selection is required to reduce fatal infections. A potential benefit of intranasal vaccination using a low virulence recombinant vaccinia virus incorporating major influenza antigens is that this strategy may circumvent pre-existing neutralizing influenza specific antibody and provide a means to efficaciously induce universal influenza immunity. Of course, we stress again that a major determinant of the successful application of any intranasal vaccination approach is that it must be accomplished with negligible inflammatory or proliferative effects in the lung, such as those described in this review.

In summary, our studies find that through histological analysis of lungs from influenza-infected mice, basic mechanisms that are associated with viral control can be observed that are not always evident in flow cytometry, ELISPOT-based studies, or other common approaches to dissect and characterize responding immune cells. While the most effective universal influenza vaccine should induce both antibody and T-cells, as synergy among immune mechanisms will provide the best protective response [5], our observations of the histological changes in the lung during primary and secondary influenza challenge highlight unique contributions to immunity that should be considered in the design of improved vaccines.

## Figures and Tables

**Table 1:** Summary of experimental models and results.

Model	Effect on T-Cells	Survival	Inflam.	BALT	Prolif.	Ref.
CD4 T Memory to WT mice	↑ CD4 T-memory	++	++	NA	+	[1]
CD4 T Memory to SCID mice	↑ CD4 T-memory	++/− *	++	0	+++ *	[1]
IL-10 Knockout mice	↑ CD8 T-cytotoxic	++	++	+	0	[6]
CCR5^−/−^CXCR3^−/−^ mice	↑ CD8 T-memory	++	++	++	++	[7]
Anti-CD25 (PC61)	↓↓ Tregs ↑ CD8 T	++	++	+++	++++	[8]

* Increase survival after clearing infection at 2 weeks, but later death from extensive proliferation. ↑ and ↓ represent increased and decreased responses, respectively.
